# The Evolution of Health Information Technology for Enhanced Patient-Centric Care in the United States: Data-Driven Descriptive Study

**DOI:** 10.2196/59791

**Published:** 2024-10-28

**Authors:** Wesley Barker, Wei Chang, Jordan Everson, Meghan Gabriel, Vaishali Patel, Chelsea Richwine, Catherine Strawley

**Affiliations:** 1 Office of the Assistant Secretary for Technology Policy US Department of Health and Human Services Washington, DC United States

**Keywords:** interoperability, e-prescribing, electronic public health reporting, patient access to health information, electronic health records, health IT

## Abstract

**Background:**

Health information technology (health IT) has revolutionized health care in the United States through interoperable clinical care data exchange, e-prescribing, electronic public health reporting, and electronic patient access to health information.

**Objective:**

This study aims to examine progress in health IT adoption and its alignment with the Office of the Assistant Secretary for Technology Policy/Office of the National Coordinator for Health IT (ASTP's) mission to enhance health care through data access and exchange.

**Methods:**

This study leverages data on end users of health IT to capture trends in engagement in interoperable clinical care data exchange (ability to find, send, receive, and integrate information from outside organizations), e-prescribing, electronic public health reporting, and capabilities to enable patient access to electronic health information. Data were primarily sourced from the American Hospital Association Annual Survey IT Supplement (2008 to 2023), Surescripts e-prescribing use data (2008 to 2023), the National Cancer Institute’s Health Information National Trends Survey (2014 to 2022), and the National Center for Health Statistics’ National Electronic Health Records Survey (2009 to 2023).

**Results:**

Since 2009, there has been a 10-fold increase in electronic health record (EHR) use among hospitals and a 5-fold increase among physicians. This enabled the interoperable exchange of electronic health information, e- prescribing, electronic public health data exchange, and the means for patients and their caregivers to access crucial personal health information digitally. As of 2023, 70% of hospitals are interoperable, with many providers integrated within EHR systems. Nearly all pharmacies and 92% of prescribers possess e-prescribing capabilities, an 85%-point increase since 2008. In 2013, 40% of hospitals and one-third of physicians allowed patients to view their online medical records. Patient access has improved, with 97% of hospitals and 65% of physicians possessing EHRs that enable patients to access their online medical records. As of 2022, three-fourths of individuals report being offered access to patient portals, and over half (57%) report engaging with their health information through their patient portal. Electronic public health reporting has also seen an increase, with most hospitals and physicians actively engaged in key reporting types.

**Conclusions:**

Federal incentives have contributed to the widespread adoption of EHRs and broad digitization in health care, while efforts to promote interoperability have encouraged collaboration across health care entities. As a result, interoperable clinical care data exchange, e-prescribing, electronic public health reporting, and patient access to health information have grown substantially over the past quarter century and have been shown to improve health care outcomes. However, interoperability hurdles, usability issues, data security concerns, and inequitable patient access persist. Addressing these issues will require collaborative efforts among stakeholders to promote data standardization, implement governance structures, and establish robust health information exchange networks.

## Introduction

The growth of health information technology (health IT) adoption and use in the United States over the years has led to better health care delivery, improved health outcomes, and enhanced patient engagement, therefore supporting patient-centric care [1]. This growth in adoption and use of health IT has been supported by numerous regulations and initiatives, including federal initiatives and legislation as well as quality improvement efforts centering around value-based care. The Health Information Technology for Economic and Clinical Health (HITECH) Act in 2009 spurred increases in the use of certified electronic health record (EHR) technology by hospitals and health care professionals [2], reflecting a collaborative effort involving public-private partnerships and various grant programs to enhance the technological foundation of health care facilities nationwide [3]. The HITECH Act was authorized by the Office of the Assistant Secretary for Technology Policy/Office of the National Coordinator for Health IT (ASTP) to develop the Health IT Certification Program and charged the Centers for Medicare and Medicaid Services (CMS) to establish the EHR Incentive Program. CMS used these programs to measure performance related to data capture and sharing for Stage 1 in 2011-2014 and performance related to clinical processes for Stage 2 in 2014 [4,5].

The enactment of the 21st Century Cures Act [6] built upon this momentum and introduced specific provisions to increase the exchange and availability of health information. This act emphasized the critical importance of interoperability and set forth measures to combat information blocking, ensuring that health care data could be exchanged and used more freely and effectively. The Cures Act final rule established further requirements to help mitigate information blocking and support health information exchange [6]. Authorized by the Cures Act, the Trusted Exchange Framework and Common Agreement (TEFCA) [7] was published in 2022, with a go-live that initiated Qualified Health Information Network applications and then designations in late 2023. TEFCA established governance, policy, and technical requirements for interoperability; connectivity to safely exchange information to improve patient care; and support for patient access to health care information. These efforts collectively focus on resolving persistent challenges for interoperability as the health care sector continues to encounter obstacles, underscoring the complexities and challenges in health IT implementation and use [8]. In 2023, the Health Data, Technology, and Interoperability: Certification Program Updates, Algorithm Transparency, and Information Sharing Rule [9] continued to implement provisions of the Cures Act to make further headway in the advancement of patient access, interoperability, and standards. Spurred on by these efforts, the advancement of health IT has brought about transformation across the US health care system.

Key advancements in health IT have paved the way for a more efficient and safer health care environment. Despite ongoing complexities, these developments have facilitated the real-time sharing of patient information among health care providers, ultimately leading to improved patient health outcomes. This paper examines the successes and challenges faced in health IT implementation and contextualizes these within the broad landscape of regulation, legislation, and initiatives put forward in the United States. Health IT, as an umbrella term, covers a wide array of technologies designed to improve health care outcomes [10]. Therefore, we focus on a broad set of technologies that have been the focus of public policymaking in support of patient-centric care: interoperable clinical care data exchange, electronic prescribing, public health reporting, and patient access to health information. This data driven analysis offers new insights by providing a critical appraisal of the health IT advances that policy has facilitated and areas where policymakers can further support patient-centric care.

## Methods

This analysis describes end-user adoption and use of health IT, focusing on interoperable clinical care data exchange capabilities, e-prescribing practices, electronic public health reporting, and patient access to electronic health information [11]. In the context of this paper, interoperable exchange is measured by the capabilities to find, send, receive, and integrate patient health information electronically for hospitals and office-based physicians. To provide a comprehensive view of the evolving health IT landscape for enhanced patient-centric care, we extracted and analyzed data from 2008 to 2023 from various sources (Table S1 in Multimedia Appendix 1).

The data for assessing interoperability and EHR adoption among hospitals were derived from the American Hospital Association (AHA) IT supplement [12] to the AHA Annual Survey, a nationally representative survey of the US hospitals. Respondents were the Chief Information Officer or the person most knowledgeable about their hospitals’ health IT capabilities. The AHA and ASTP have collaborated to monitor the adoption and use of health IT in the US hospitals since 2008. Annual response rates to the IT supplement vary, but average greater than 50% of nonfederal acute care hospitals in the US. Nonfederal acute care hospitals were the focus of our analyses due to their receipt of EHR incentive payments.

The data for assessing interoperability and EHR adoption among physicians were derived from the Centers for Disease Control and Prevention’s (CDC) National Center for Health Statistics’ National Electronic Health Records Survey [13,14]. These data span from 2009 to 2021 and track trends among nonfederally employed office-based physicians in adopting and using EHRs for direct patient care. This survey excludes nondirect care providers such as radiologists and pathologists and provides valuable insights into the EHR functionalities used in outpatient settings. Annual responses to the survey vary. In 2021, a total of 1875 responses were weighted to reflect national estimates for approximately 403,013 office-based physicians in the United States.

For e-prescribing trends, Surescripts transactional data from 2008 to 2023 [15-17], a prominent e-prescription network in the United States, were used. This network captures data across a wide array of pharmacies, and is used by a majority of chain, franchise, or independently owned pharmacies in the US. Surescripts routed 134 million e-prescribing messages in 2008 and 2.5 billion in 2023.

The National Cancer Institute's Health Information National Trends Survey (HINTS) [18] were used to measure engagement with health IT, tracking how people access and use health information, their use of information technology for health management, and their engagement level in health-related behaviors. This nationally representative survey offers insights into public engagement with health IT and the evolving trends in this domain. The survey has been fielded since 2003; data used for this analysis are from 2014-2022. Data were collected from 3677 respondents in 2014 and 6252 respondents in 2022, and responses were weighted to generate national estimates.

The University of California San Francisco (UCSF) National Physician Health IT Survey was used to measure primary care physicians who reported to immunization information services (IIS) through their EHR. This nationally representative survey of office-based physicians was conducted in partnership with the UCSF, ASTP, and the American Board of Family Medicine in 2022 [19,20]. Data from 1375 respondents specializing in family medicine, internal medicine, obstetrics and gynecology, pediatrics, and other primary care specialties and whose email addresses were listed in the Definitive Healthcare data were collected. Analyses focused upon physicians who used EHRs and who worked in primary care for nonfederal employers. Responses were weighted to generate national estimates.

The CMS established the Promoting Interoperability (PI) Program (formerly the EHR Incentive Program) in 2011 to encourage certain providers and hospitals to adopt, use, and demonstrate the meaningful use of certified electronic health record technologies [21]. These data were used to estimate hospital and physician adoption of electronic capabilities and enablement to submit immunizations, syndromic surveillance, and laboratory reports to public health jurisdictions.

## Results

### Overview

**Figure 1:**
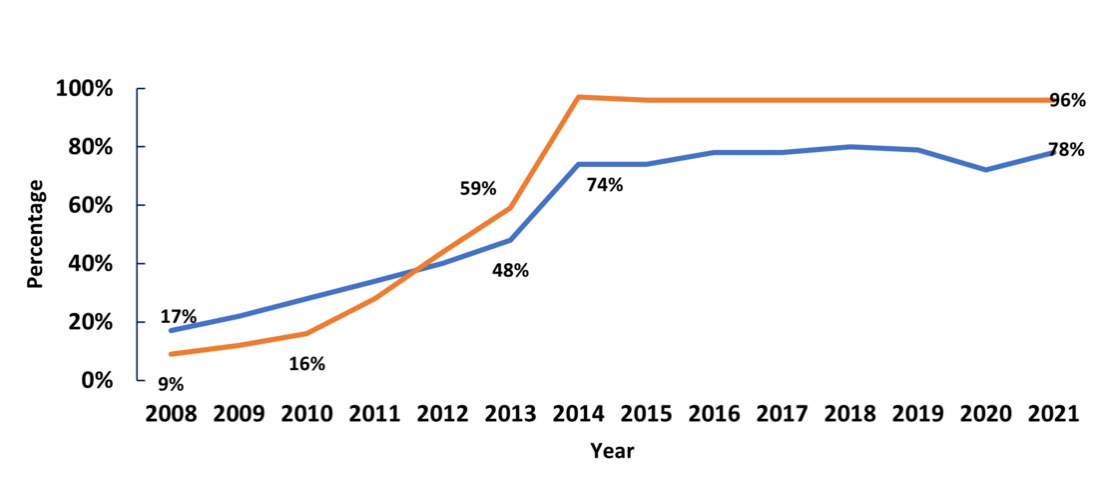
Electronic health record adoption among hospitals and physicians. Blue line: Percent of office-based physicians; orange line: Percent of nonfederal acute care hospitals. Sources: American Hospital Association Information Technology Supplement (2008-2021) and National Center for Health Statistics Ambulatory Care Survey (2008-2011) and National Electronic Health Record Survey (2012-2021).

Many physicians and hospitals benefitted from federal incentive payments, authorized by the HITECH Act, to implement and use certified health IT, shown by the surge in certified health IT adoption among office-based physicians and nonfederal acute care hospitals. EHR adoption and use, underpinning interoperable clinical care data exchange, e-prescribing, electronic public health reporting, and patient access to health information increased over the past 2 decades. Since 2008 there was an 87%-point increase in EHR use among hospitals and a 61%-point increase among physicians (Figure 1).

In Figure 1, 2008-2013 includes hospitals and physicians that have adopted an EHR system that integrates patient data, medication tracking, clinician notes, and diagnostic results. 2014 to 2021 includes hospitals and physicians that have adopted a certified EHR system that meets the capability, functionality, and security requirements adopted by the US Department of Health and Human Services. This graph represents data collected from office-based physicians and nonfederal acute care hospitals.

### Interoperable Clinical Care Data Exchange

**Figure 2:**
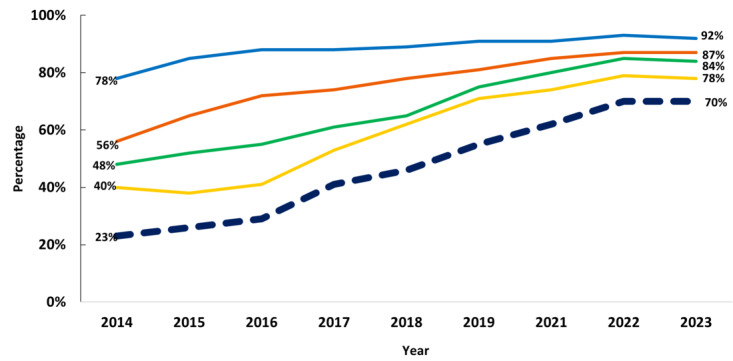
Hospitals engaging in interoperable exchange of electronic health information. Blue line: Send; orange line: Receive; green line: Find; yellow line: Integrate; Blue dotted line: Hospital engagement in all 4 domains of interoperability. Source: 2014-2023 American Hospital Association Information Technology Supplement. These data were collected from nonfederal acute care hospitals.

Following this period of rapid digitization, there has been progress related to interoperable data exchange among hospitals (Figure 2). From 2014 to 2023, the engagement in all 4 interoperable exchange domains (find, send, receive, and integrate patient health information electronically) increased. During this decade, the ability to find information increased by 75%, from 48% in 2014 to 84% in 2023. The ability to send information increased by 18%, from 78% in 2014 to 92% in 2023. The ability to receive information increased by 55%, from 56% in 2014 to 87% in 2023. Finally, the ability to integrate information increased by 95%, from 40% to 78% in 2023. In 2014, 23% of hospitals were engaged in all 4 interoperable exchange domains either routinely or sometimes. By 2023, 70% of hospitals sometimes or routinely engaged in all 4 interoperable exchange domains. Notably, while over half of hospitals were not fully interoperable in 2018, by 2023, this declined to 30%, indicating an improvement in interoperable exchange among hospitals.

**Figure 3:**
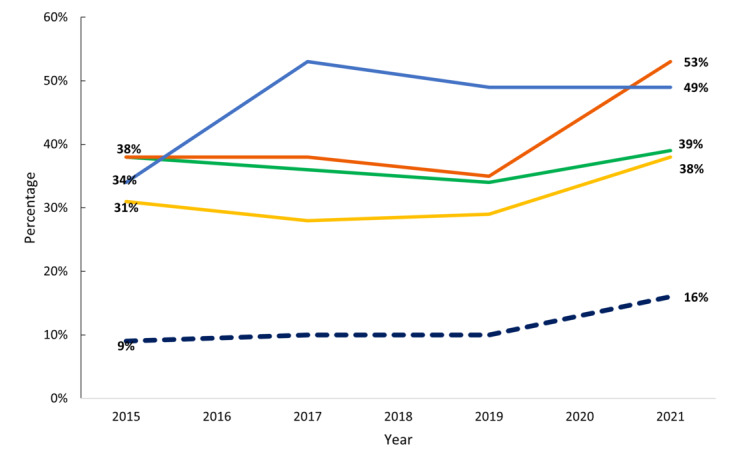
Physicians Engaging in Interoperable Exchange of Electronic Health Information. Green line: Send; orange line: Receive; blue line: Find; yellow line: Integrate; blue dotted line: Physician engagement in all 4 domains of interoperability. Source: 2015-2021 National Electronic Health Record Survey. These data are collected from office-based physicians.

For office-based physicians, there has been modest progress related to interoperable exchange (Figure 3). From 2015 to 2021, like hospitals, office-based physicians’ engagement in all 4 interoperable exchange domains increased, most notably, the abilities to find and receive electronic information. During this 7-year period, the ability to find information increased by 44%, the ability to receive information increased by 39%, the ability to send information increased by 3%, and the ability to integrate information increased by 23% among office-based physicians. By 2021, 16% of office-based physicians were engaged in all 4 interoperable exchange domains, with a 78% increase in engagement from 2015 to 2021.

### Electronic Prescribing

**Figure 4:**
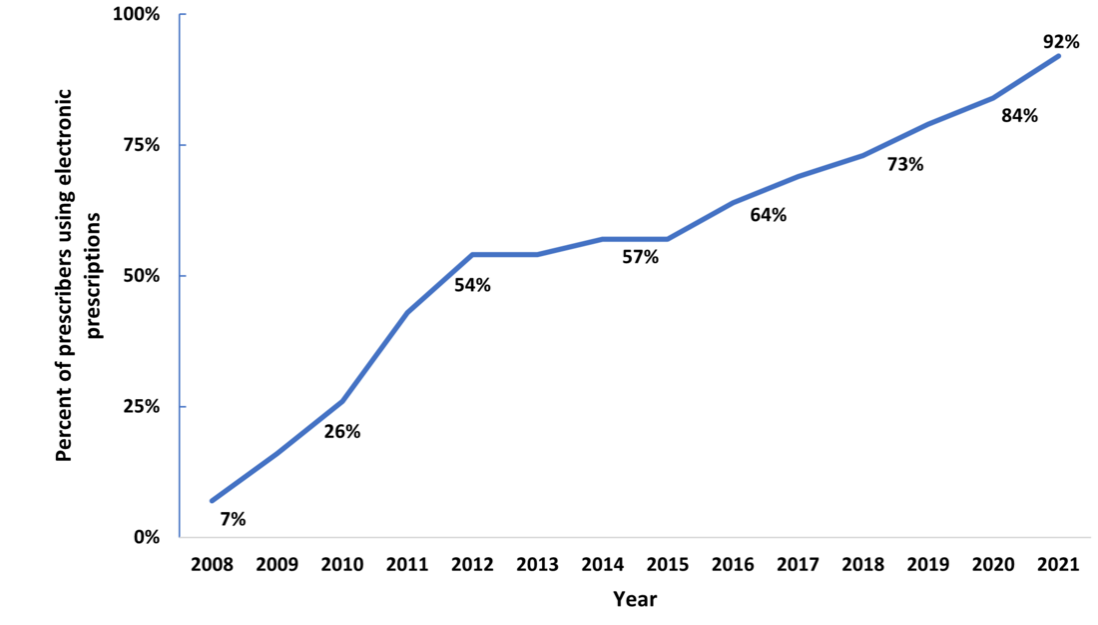
Electronic prescribing use among prescribers. Source: Analysis of Electronic Prescribing Transaction Data.

**Figure 5:**
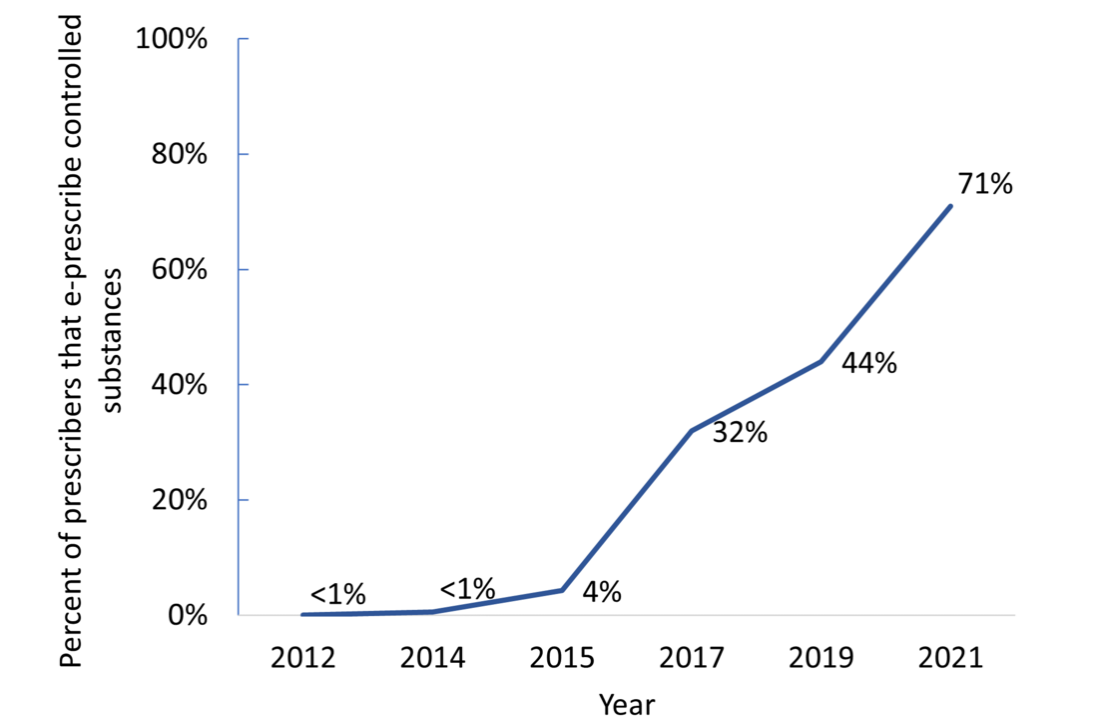
Electronic prescribing of controlled substances. Sources: EPCS (electronic prescribing of controlled substances) Transactional Data (2012-2015, prescribers enabled for ECPS) and National Center for Health Statistics Ambulatory Care Survey National Electronic Health Record Survey (2017-2021, percent of office-based physicians using EPCS).

The percentage of prescribers who e-prescribe has grown from 7% in 2008 to 92% in 2021, an 85%-point increase. Now, there is near-universal adoption among pharmacies, and 92% of prescribers are enabled for e-prescribing (Figure 4). In addition, the electronic prescribing of controlled substances (EPCS) has increased since first allowed in 2010 (Figure 5). Early on, uptake of EPCS was slow, from 0.05% in 2012 to just over 4% in 2015. By 2017, about a third of office-based physicians who prescribe were using EPCS, and as of 2021, nearly three-fourths of them were using EPCS.

### Electronic Public Health Reporting

Public health reporting capabilities among hospitals and primary care physicians have increased over the past decade (Textbox 1). An analysis of CMS’s Promoting Interoperability Program data shows that in 2012, among eligible hospitals required to use certified health IT to report public health data electronically, 63% could electronically submit immunization data to their state IIS, 57% could electronically report laboratory results, and 55% could electronically report syndromic surveillance data to public health agencies. For primary care physicians participating in the program, over half (57%) were able to electronically submit immunization data to their state IIS.

**Public health reporting among hospitals and physicians.**

**2012**

- 63% of participating hospitals enabled to report immunization data to public health agencies.
- 57% of participating hospitals enabled to report laboratory results to public health agencies.
- 55% of participating hospitals enabled to report syndromic surveillance data to public health agencies.
- 57% of participating primary care physicians enabled to report to immunization information services.

**2022**

- 90% of hospitals enabled to report immunization data to public health agencies.
- 85% of hospitals enabled to report laboratory results to public health agencies.
- 86% of hospitals enabled to report syndromic surveillance data to public health agencies.
- 74% of primary care physicians who viewed immunization data in their electronic health record report data to immunization information services.

By 2022, according to the AHA Health IT Supplement, rates of hospitals’ electronic reporting increased for syndromic surveillance (86%) and laboratory reporting (85%) and immunization registry reporting (90%). In addition, as of 2022, about three-fourths of primary care physicians who viewed immunization data in their EHR indicated their primary outpatient EHR reported data to their state IIS.

### Patient Access to Health Information

**Figure 6:**
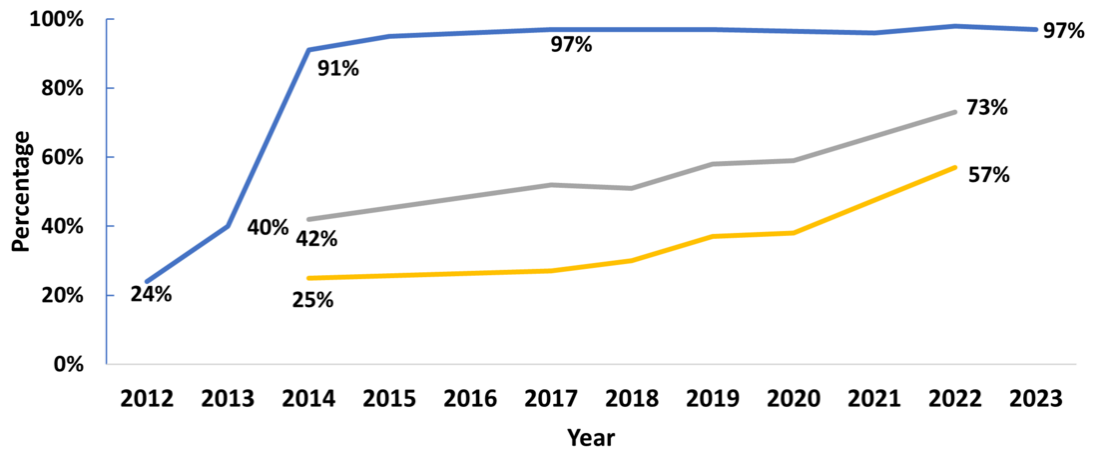
Access to patient electronic health information. Blue line: Hospital capability to enable patient electronic access to health information. Sources: NEHRS (National Electronic Health Record Survey), AHA (American Hospital Association) IT Supplement, and HINTS (Health Information National Trends Survey) Surveys. Blue line: Hospital capability to enable patient electronic access to health information, grey line: Individuals offered a patient portal by a provider or insurer, yellow line: Individuals offered and accessed a patient portal.

Over the past decade, progress has been made in enabling patient access to electronic health information (Figure 6). In 2012, 24% of nonfederal acute care hospitals offering inpatient care had health IT capable of enabling patients to view their online medical records. As of 2017, 97% of nonfederal acute care hospitals providing inpatient care possessed health IT that enabled patients to view their electronic health information online, predominantly through patient portals or smartphone health apps. This percentage has remained stable throughout the subsequent years, and nearly all (97%) of hospitals offering inpatient care in 2023 enabled patient access to their data.

In 2014, 42% of individuals were offered a patient portal by their provider or insurer. In 2022, 73% of individuals reported being offered online access to their medical records by health care providers or insurers, representing a 74% increase since 2014. Only a quarter of individuals were offered and accessed their data in 2014. However, by 2022, the percentage of individuals who were offered and access their online medical records rose to 57%, a 128% increase.

## Discussion

### Principal Findings

Our analysis provides insights into the outcomes of ongoing efforts to enhance health care delivery through advanced technology and data exchange. High adoption rates for health information technologies among health care professionals and hospitals since 2009 underscore the critical role of federal incentives and collaborative efforts [22-24]. We also highlight the need for continued efforts to overcome the challenges in health IT implementation and ensure that all patients have access to high-quality, patient-centric care. The rise in the adoption of these functionalities reflects the success of initiatives and programs designed to transition health care from mostly paper-based to digitally native. The rise in the adoption of these functionalities reflects a broader trend toward greater patient involvement in their health and care.

### Interoperable Clinical Care Data Exchange

The growth in interoperable exchange among health care providers signifies a transformative era in health care communication and data exchange [25]. These findings show that both hospitals and office-based physicians alike have made progress in interoperable exchange and the adoption and use of health IT. However, office-based physicians have consistently lagged behind hospitals. With the modest progress in interoperable exchange among physicians, it is important to continually monitor growth to inform targeted initiatives and support. Furthermore, previous analyses examining variation in interoperable exchange across both hospitals and physicians show that providers with greater resources outpace those with fewer resources, and the latter group continue to face challenges that need to be addressed [26-28].

Despite marked growth in interoperable exchange among hospitals, the majority of hospitals continue to experience interoperability challenges, and lower-resourced hospitals reported challenges with using application programming interfaces (APIs) for more efficient information exchange [29]. Also, more than half of physicians experienced positive benefits from engaging in electronic exchange of patient health information in areas related to practice quality, care coordination, and efficiencies, yet most physicians also experienced barriers to exchange [30]. Although, a recent survey of family medicine physicians revealed that the majority (91%) find it at least somewhat easy to use external information for patient care, around 8 out of 10 reported facing challenges in locating important information and integrating it into their EHR. These data point to the need to explore interventions that support data integration to ensure that accessing and using patient information from external sources becomes seamless.

Despite the investments of the past 2 decades, more work to enable clinical data exchange remains. Of particular importance is data standardization and the seamless exchange of information across diverse health systems. The continuing evolution of technology and legislative and regulatory support are essential to address these barriers and advance interoperable exchange, as this is a critical factor in the integration and advancement of health IT, allowing health care providers to share patient information in real time, leading to improved patient outcomes [31,32]. In addition, necessary information is not always available at the point of care and hospitals may not always be able to share patient information with external providers including other hospitals, ambulatory care, long-term care providers, or behavioral health, even when they have the capability [33]. This can lead to providers frequently missing patient records, as a recent survey of family medicine providers found that a third frequently encountered this issue [28]. Improvements that come with enhanced interoperability will play a role in providing patient information that is needed and when it is needed (at the point of care).

### E-Prescribing

The rise in e-prescribing adoption illustrates another shift toward digital health care processes to help improve prescription accuracy, efficiency, and patient safety [27,34]. Research suggests that adherence to early e-prescribing thresholds set forth by the Medicare Promoting Interoperability Program is associated with reduced rates of adverse drug events, indicating the potential of e-prescribing to improve medication accuracy and patient adherence [35]. The integration of e-prescribing into EHRs and the advancement of EPCS have been crucial in this development. The use of e-prescribing is now nearly universal, and the use of EPCS [36] is growing rapidly, and being facilitated by state and federal initiatives [37] and the integration of systems within EHRs.

However, there are still areas for improvement, particularly in ensuring uniform adoption across all health care settings. Future efforts must focus on overcoming these challenges to fully realize the benefits of e-prescribing in enhancing patient care. The use of EPCS has contributed to safer prescribing practices and aiding in combating the opioid crisis. Also, prescription drug monitoring programs [37] (PDMPs) have become integral tools in addressing the opioid epidemic, offering real-time data to aid health care providers in making informed prescribing decisions. While PDMPs have shown promise in reducing inappropriate prescribing, their effectiveness is amplified when integrated with EHRs, highlighting the importance of ease of access and use for health care professionals [38-40]. Recent studies confirmed that physicians frequently use PDMPs [37] and report benefits beyond reduced prescribing, including improved clinical decision-making and overall patient care. The growth of EPCS and other related tools and initiatives, such as PDMPs, have affected improvement on patient-centric care by reducing inappropriate [41] controlled substance prescribing practices and prescription overdoses. While the adoption and use of e-prescribing of medications inclusive of controlled substances has been hailed as a success, there is still room for improvement. Updating the National Council for Prescription Drug Programs Script standards to align with Medicare and other federal programs would help to ensure a unified approach across our health care system [42]. This alignment would help to ensure that these tools enhance patient care function optimally and provide accurate, efficient, and compliant e-prescribing and monitoring of controlled substances.

Furthermore, to reduce the burden associated with medication before approvals, the Health Information Technology Advisory Committee [43] Taskforce on Pharmacy Interoperability and Emerging Therapeutics [44] has recommended that electronic previous authorizations be included with Health IT Certification requirements and to support better decision-making during prescribing. The implementation of real-time benefit tools [45] is important, as reflected by legislative requirements [46], and will help improve prescription affordability and transparency. To further enhance patient-centric care, it may be beneficial for providers and pharmacies to include additional data elements, such as the indication or reason why a medication was prescribed, on e-prescriptions [47]. This will improve communication between the pharmacist and patient regarding the intended use of the medication and ensure safe and effective care. These updates and improvements to e-prescribing are set to transform it into a more integrated tool for medication management in support of patient-centric care. It would help to improve bidirectional access between parties, uphold patient privacy and security, and address existing gaps in prescription services.

### Electronic Public Health Reporting

The adoption of electronic public health reporting capabilities for both hospitals and physicians has changed the landscape of public health surveillance and response, driven by legislative frameworks such as the HITECH Act and furthered by the CMS PI Programs [26,48-50]. The transition from optional to mandatory reporting [51] on public health measures for providers under these programs has increased electronic public health reporting capabilities, streamlining the submission of public health data, and equipping public health agencies with more timely and accurate data [52] needed for disease surveillance and response efforts [2].

Despite this progress, providers continue to face challenges to electronic public health reporting due to a lack of data standardization and use of different vocabulary standards, high implementation costs related to interfaces and data submission or transmission, technical complexity of interfaces, and difficulty extracting relevant information from the EHR [50]. Furthermore, many providers cite public health agencies’ lack of capacity to electronically receive information as a major barrier to public health information exchange [26,53]. Varying technical complexities of sending and receiving systems across institutions persist, highlighting a need for comprehensive strategies to enhance system uniformity and functionality to achieve interoperable public health data systems. Nevertheless, the ongoing digitization of public health reporting has facilitated a notable improvement in data exchange and real-time monitoring capabilities.

Public health reporting is crucial for rapid response and effective public health management, as evidenced by the increased adoption rates among hospitals for electronic submission of immunization, syndromic surveillance, and laboratory data and among physicians for IIS reporting efforts. A similar trend is seen among physicians, among whom there was early uptake of exchange, and rates have remained steady since then [19,54]. Recent data show that around half of primary care physicians exchange information with their state’s IIS, but among those who viewed immunization data in their EHR, approximately three-fourths of them were able to access and report data to their IIS [19]. Rates of awareness of their EHR’s reporting capabilities and overall satisfaction among primary care physicians varied, and the data show that those physicians that exchanged with their IIS through their primary outpatient EHR have a higher rate of satisfaction than those using an outside portal, paper, or fax, showing the perceived benefits of integrating these capabilities within the EHR [19].

Federal mandates to report public health data electronically and underlying Health IT Certification Program requirements that standardize these processes across disparate health IT have played a role in advancing electronic reporting. The mandates led to an uptake in electronically reporting key public health data, underscoring the influence of federal policy in enhancing public health reporting infrastructures. Despite advancements driven by federal requirements and coordination with state, tribal, local, and territorial health departments, interoperable exchange issues and the integration of reporting functionalities within existing health IT present ongoing challenges, highlighting the need for continued collaboration among stakeholders to foster a more integrated and efficient public health reporting ecosystem.

Though progress has been made in electronic public health reporting, addressing existing challenges remains critical for further developing efficient, standardized, and universally accessible public health reporting systems. Enhancing electronic reporting capabilities across all health care settings, particularly among office-based physicians, is essential for achieving comprehensive public health surveillance and ensuring a coordinated response to public health emergencies. These challenges underscore the importance of continued support and resource allocation from federal initiatives to address underlying barriers to the interoperable exchange of public health data. The CDC’s Data Modernization Initiative is one such effort aimed at modernizing public health infrastructure needed to facilitate the flow of timely, actionable data to inform public health decision-making and developing a trained public health workforce to support evolving data needs. The CDC’s Public Health Data Strategy highlights specific areas of focus for Data Modernization Initiative investment and outlines specific actions and goals, including strengthening the core of public health data and advancing open and interoperable public health data, to ensure critical core data elements can be shared between health care providers and public health [55].

### Patient Access to Electronic Health Information

In recent years, advancements in health IT have notably enhanced patient access to electronic health information, underpinned by provisions from the 21st Century Cures Act to reduce the effort for patients to electronically access their health information and increased demand due to the COVID-19 pandemic’s impact on health care delivery. The transition to EHRs facilitated by federal mandates fostered an informed and engaged patient population. The adoption of certified health IT has not only streamlined patient engagement but also enhanced the quality of health care delivery. The surge in telehealth and patient portal usage from 2020 to 2022 [56] exemplifies the role that external factors, such as the COVID-19 pandemic, have played in accelerating the demand for electronic access to health information. As of 2022, most individuals who accessed their data did so to view test results, view clinical notes, or download health information [56]. This period underscored the use of patient portals and health applications in providing crucial health care services and information, thereby contributing [57] to an increase in their adoption and use.

These developments signify a pivot toward more patient-centric health care, with increased patient engagement and demand for transparent, accessible health information [58]. Data indicate that nearly all hospitals report the ability to enable patients to access their information online. Expanded health IT adoption, however, illuminates persistent access and usage differences across different patient groups, accentuated by varying levels of education, income, and ethnicity [59]. These differences highlight the critical need [60] for targeted interventions to bridge gaps and ensure that advancements in health IT benefit all sections of the population equitably. Recent data show that family physicians who practiced in rural areas, that did not have staff and linkages to community programs to address patients’ social needs, and that treated a large proportion of vulnerable patients were less likely to be satisfied with access to patient information from external providers [28]. Therefore, the inability to access and use information from external sources creates friction and barriers to patient-centric care.

The shift toward app-based access to health information introduces new dimensions to the health IT landscape, offering increased convenience and patient engagement [56]. However, this shift [61] also raises new challenges when it comes to data privacy, security, and interoperability. As mandated by the Cures Act Final Rule [6], implementing standards-based APIs represents a step forward in addressing these challenges, promoting safer and more efficient access to health information. Addressing these multifaceted challenges and opportunities is imperative for advancing a more inclusive, effective, and patient-centric health care system.

### Raising the Bar to Support Patient-Centric Care

Ultimately, health IT has contributed to more patient-centric care in the United States [62], but more work remains to ensure that information is exchanged across the health care delivery system and that exchanged information is easily usable by humans and machines. Enhancing standards for data sharing, expanding transparency and accountability, promoting open APIs, addressing information blocking, and leveraging lessons from the COVID-19 pandemic will help to further facilitate and support patient-centric care.

For example, data standards, such as the United States Core Data for Interoperability [63], which was established in the Cures Act Final Rule [64], includes data elements, classes, and standard code set versions that are continuously and transparently updated through an annual, public process. The implementation of these updated standards and frameworks are integral to support seamless exchange of data elements needed for patient-centric care. In addition, the Health Data, Technology, and Interoperability: Certification Program Updates, Algorithm Transparency, and Information Sharing Rule supports provisions from the Cures Act and sets first of its kind transparency requirements for artificial intelligence used in health IT [9]. The establishment of TEFCA, and the adoption of secure, standardized APIs that can be accessed and used without special effort will help to facilitate and support necessary data sharing to promote patient-centric care.

Since the start of this decade, federal policymakers have focused on initiatives and policies to address persistent challenges with interoperable exchange. The implementation of provisions of the 21st Century Cures Act [65], such as defining information blocking, establishing disincentives, and clarifying valid exceptions are initiatives to help support interoperable exchange [64,66]. In addition, insights from the COVID-19 pandemic tell us that although interoperable exchange has not been fully achieved, the rapid adoption and implementation of new digital health and virtual care models, which were vital during the pandemic, were made possible. Due to this, rates of patient engagement and electronic public health reporting have improved, and further advancements to interoperability and transparency through standards and policies that support the access, use, and exchange of patient information are helping to move the needle forward.

As technology advances, it is essential to update and standardize the way health IT applications launch and interact. A specific focus on security, data accessibility, and interoperable exchange will help to ensure that patients can access their electronic health information. These efforts are meant to broaden the playing field, inviting innovators and new models for data sharing, clinical decision-making, and patient access to their electronic health information. The rapid digitization of the past 2 decades has created new opportunities in health care technology and delivery.

This analysis provides a high-level overview of advancements in health IT that have revolutionized patient-centric care in the United States. Continuous measurement and evaluation of health IT policy and program implementation have been vital to identify barriers and help formulate ways to address these challenges. Due to the complex nature and inherent difficulties in quantifying interoperable exchange among physicians, it is important to explore methods for accurate measurement and to address these challenges. This will enable more comprehensive evaluations of interoperability. Furthermore, advancement of indices or tools to holistically measure interoperability and related capabilities could be used to assess the impacts of policies, inform future policy opportunities, and identify areas for targeted support, including performance gaps and health equity concerns. As we move forward into the next quarter-century, it is now the time to be bold and help support tools and processes to ensure patient-centric care [67].

### Conclusion

The United States has experienced growth in enhanced information exchange, e-prescribing, electronic public health reporting, and patient access to health information, which have transformed health care delivery and improved patient outcomes. While these achievements are noteworthy, it is essential to continue the collaborative efforts, policy support, and technological innovation to overcome the persistent obstacles, such as nonstandardized health IT systems and financial burdens. Legislation such as the HITECH Act and 21st Century Cures Act, have played a vital role in addressing these challenges.

Addressing social determinants and ensuring equitable technology access remain central to bridging the digital divide and achieving a comprehensive health care transformation. In the next 25 years, we should shift our focus from health IT adoption to infrastructure improvements necessary for patient-centric care. It is crucial to establish a system where patient data can flow safely and seamlessly. The commitment to advancing health IT will be indispensable in overcoming existing challenges and harnessing the full potential of technological innovations in health care as we transition toward a more integrated and patient-centric health care framework. The ongoing evolution of health IT offers a future where health care delivery is efficient, accessible, and tailored to the needs of every patient, creating another quarter-century of improvements in public health and patient care.

## Appendix

Multimedia Appendix 1 Data sources.

## Abbreviations

AHA: American Hospital Association
API: application programming interface
ASTP: Office of the Assistant Secretary for Technology Policy/Office of the National Coordinator for Health IT
CDC: Centers for Disease Control and Prevention
CMS: Centers for Medicare and Medicaid Services
EHR: electronic health record
EPCS: electronic prescribing of controlled substances
HITECH: Health Information Technology for Economic and Clinical Health
IIS: immunization information service
PDMP: prescription drug monitoring program
PI: Promoting Interoperability
TEFCA: Trusted Exchange Framework and Common Agreement
UCSF: University of California San Francisco
