# Supplementary material for: The Evolution of Health Information Technology for Enhanced Patient-Centric Care in the United States: Data-Driven Descriptive Study
Source: J Med Internet Res. 2024 Oct 28;26:e59791. doi: 10.2196/59791 (PMC11555447; doi:10.2196/59791)
Supplement: Multimedia Appendix 1 [file jmir_v26i1e59791_app1.docx]

**Table S1.** Data Sources.

| Organization | Data Source | Sampling Frame | Years of Data Used |
| --- | --- | --- | --- |
| American Hospital Association | American Hospital Association (AHA) Annual Survey and Information Technology Supplement | Hospitals in the United States | 2008 -2024 |
| National Center for Health Statistics | National Electronic Health Records Survey (NEHRS) from 2012-2022 and the National Center for Health Statistics Ambulatory Care Survey (NAMCS) from 2008-2011 | Non-federally employed, office-based physicians in the United States | 2008-2021 |
| Surescripts | Surescripts Transactional Electronic Prescribing Data | Electronic prescriptions routed through the Surescripts Network | 2008-2023 |
| National Cancer Institute | Health Information National Trends Survey (HINTS) | Individuals in the United States | 2014-2022 |
| American Board of Family Medicine, University of California, San Francisco, and The Office of the National Coordinator for Health IT | National Physician Health IT Survey | Office-based physicians in the United States | 2022 |
| Centers for Medicare and Medicaid Services | Promoting Interoperability (PI) Program Data (formerly the EHR Incentive Program) | Eligible professionals and eligible hospitals | 2012 |
